# High-demand tasks show that ACL reconstruction is not the only factor in controlling range of tibial rotation: a preliminary investigation

**DOI:** 10.1186/s13018-023-03639-2

**Published:** 2023-03-13

**Authors:** Mark Zee, Michele Keizer, Jos van Raaij, Juha Hijmans, Inge van den Akker-Scheek, Ron Diercks

**Affiliations:** 1 Department of Orthopaedic Surgery, University of Groningen, University Medical Center Groningen, Hanzeplein 1, PO Box 30.001, 9700 RM Groningen, The Netherlands; 2 Department of Human Movement Science, University of Groningen, University Medical Center Groningen, Hanzeplein 1, PO Box 30.001, 9700 RM Groningen, The Netherlands; 3 Department of Orthopaedic Surgery, Martini Hospital, Van Swietenplein 1, 9728 NT Groningen, The Netherlands; 4 Department of Rehabilitation Medicine, University of Groningen, University Medical Center Groningen, Hanzeplein 1, PO Box 30.001, 9700 RM Groningen, The Netherlands

**Keywords:** Anterior cruciate ligament injury, Motion capture system, In vivo analysis, Range of tibial rotation, Knee

## Abstract

**Background:**

Excessive range of tibial rotation (rTR) may be a reason why athletes cannot return to sports after ACL reconstruction (ACLR). After ACLR, rTR is smaller in reconstructed knees compared to contralateral knees when measured during low-to-moderate-demand tasks. This may not be representative of the amount of rotational laxity during sports activities. The purpose of this study is to determine whether rTR is increased after ACL injury compared to the contralateral knee and whether it returns to normal after ACLR when assessed during high-demand hoptests, with the contralateral knee as a reference.

**Methods:**

Ten ACL injured subjects were tested within three months after injury and one year after reconstruction. Kinematic motion analysis was conducted, analysing both knees. Subjects performed a level-walking task, a single-leg hop for distance and a side jump. A paired t-test was used to detect a difference between mean kinematic variables before and after ACL reconstruction, and between the ACL-affected knees and contralateral knees before and after reconstruction.

**Results:**

RTR was greater during high-demand tasks compared to low-demand tasks. Pre-operative, rTR was smaller in the ACL-deficient knees compared to the contralateral knees during all tests. After ACLR, a greater rTR was seen in ACL-reconstructed knees compared to pre-operative, but a smaller rTR compared to the contralateral knees, even during high-demand tasks.

**Conclusion:**

The smaller rTR, compared to the contralateral knee, seen after a subacute ACL tear may be attributed to altered landing technique, neuromuscular adaptation and fear of re-injury. The continued reduction in rTR one year after ACLR may be a combination of this neuromuscular adaptation and the biomechanical impact of the reconstruction.

*Trial registration:* The trial was registered in the Dutch Trial Register (NTR: www.trialregister.nl, registration ID NL7686).

**Supplementary Information:**

The online version contains supplementary material available at 10.1186/s13018-023-03639-2.

## Background

In the population of young athletes, return to sports after ACL reconstruction (ACLR) has become an increasingly relevant outcome. A review of literature shows that a mere 55% of athletes can return to a competitive form of sports after ACLR [1]. Historically, reconstruction techniques have focused on restoring anterior tibial translation (ATT). However, it is known that the ACL also plays an important role in limiting tibial rotation [2]. Excessive tibial rotation can potentially lead to giving way. This persistent feeling of giving way may be a reason why athletes cannot perform at their pre-injury level of sports.

Tibial rotation has so far been measured during low-to-moderate-demand tasks (e.g. walking, cutting, pivoting). Increased tibial rotation is demonstrated in chronic ACL deficiency compared to healthy knees. After ACLR, decreased rTR compared to healthy knees has been shown [3–16]. Decreased tibial rotation after ACLR does not comply with a potential persistent feeling of giving way after ACLR. One reason might be, that up to now, subjects have not been tested under sports related circumstances. While cutting and pivoting are considered relevant for sports activities, hoptests have the potential to test the combination of eccentric and concentric power and strength and neuromuscular coordination and knee stability [17]. We consider the fact that patients experience more rotational instability during high-demand activities like jumping, ultimately hampering return to sports rates.

Successful performance on a battery of hop tests is recommended as one of the criteria for return to sports, as these tasks simulate high-demand activities during pivoting sports, albeit in a controlled environment [18–20]. Measuring tibial rotation during hop tests using motion capture systems may provide more insight into knee kinematics during return-to-sports activities.

We hypothesize that range of tibial rotation (rTR) is greater in the ACL deficient knee compared to the contralateral intact knee and remain similar after ACLR when measured during high-demand functional tasks, replicating sports activities, while a decrease is seen during low-demand tasks, as is seen in previous studies. This study aims to determine rTR before and after ACLR, assessed during low- and high-demand functional tests.

## Methods

### Design

This trial was set up as a multicentre prospective cohort study. Martini Hospital and University Medical Center Groningen (UMCG), both large teaching hospitals, served as recruiting centers. The study protocol was reviewed and approved by the Institutional Review Board of UMCG (registration ID 2015/524, UMCG trial Register No. 201501098). The trial was registered in the Dutch Trial Register (NTR: www.trialregister.nl, registration ID NL7686).

### Participants

From June 2016 to June 2018 all patients diagnosed with ACL injury in one of the participating hospitals were consecutively screened for eligibility to participate in the study. Inclusion criteria were: (1) age 18–35 years, (2) unilateral ACL rupture confirmed by physical examination, (3) less than three months post-injury at time of diagnosis, (4) at least six weeks of conservative therapy, (5) intact contralateral knee on physical examination, (6) absence of concomitant injury to cartilage, bone, meniscus or other ligaments on MRI. Exclusion criteria were: (1) any history of fractures, osteotomy or previous ligament reconstructive surgery in the lower extremities or spine, (2) neurological conditions leading to musculoskeletal disorders, (3) any other musculoskeletal pathology of the lower limbs (i.e. concomitant ligamental injuries or meniscal injuries), (4) inability to complete questionnaires in Dutch.

As presence or absence of any concomitant knee injury can influence the degree of tibial rotation; as injury to the menisci and anterolateral structures of the knee are known to play a role [21], we only included subjects without concomitant injury to the knee.

Conservative therapy prior to testing was initiated upon diagnosis and consisted of physiotherapy sessions at least 2 times per week. Pre-rehabilitation was performed according to the Dutch guideline on ACL injury and focused on decrease of effusion, increase of range of motion and quadriceps and hamstrings strengthening exercises.

### Surgical procedure

All subjects underwent anatomic, single-bundle ACLR using a semitendinosus/gracilis graft as part of usual care. Both tendons were doubled to create a four-strand graft. The femoral tunnel was created independent of the tibial tunnel via an anteromedial portal technique. For femoral fixation a suspension type fixation was used (Endobutton, Smith&Nephew, London, UK). After pretensioning (60N), tibial fixation was performed by using a PEEK screw and plug (Biosure PK, Smith&Nephew, London, UK). Surgical procedures were performed by two orthopaedic surgeons experienced in ACLR. Surgeon allocation was dependent on site of inclusion.

### Motion data collection

The motion data collection was performed at the motion lab of UMCG’s Department of Rehabilitation Medicine. The motion lab consists of a 9 m walkway with two 40 × 60 cm force plates (AMTI; Watertown, MA, USA) embedded in the floor. An 8-camera optoelectronic motion capture system (VICON MX, Vicon Motion Systems Ltd., Oxford, UK) sampling at 100 Hz was used. The position of 22 14 mm spherical markers distributed on the lower extremities according to Hayes and Davis was recorded [22]. Marker placement was performed by the same researcher during this study. After static and dynamic calibration, joint centres were calculated using VICON Nexus software v2.8 (VICON MX, Vicon Motion Systems Ltd., Oxford, UK). For the complete procedure and its sensitivity, see Keizer and Otten (2020) [23].

**Fig. 1:**
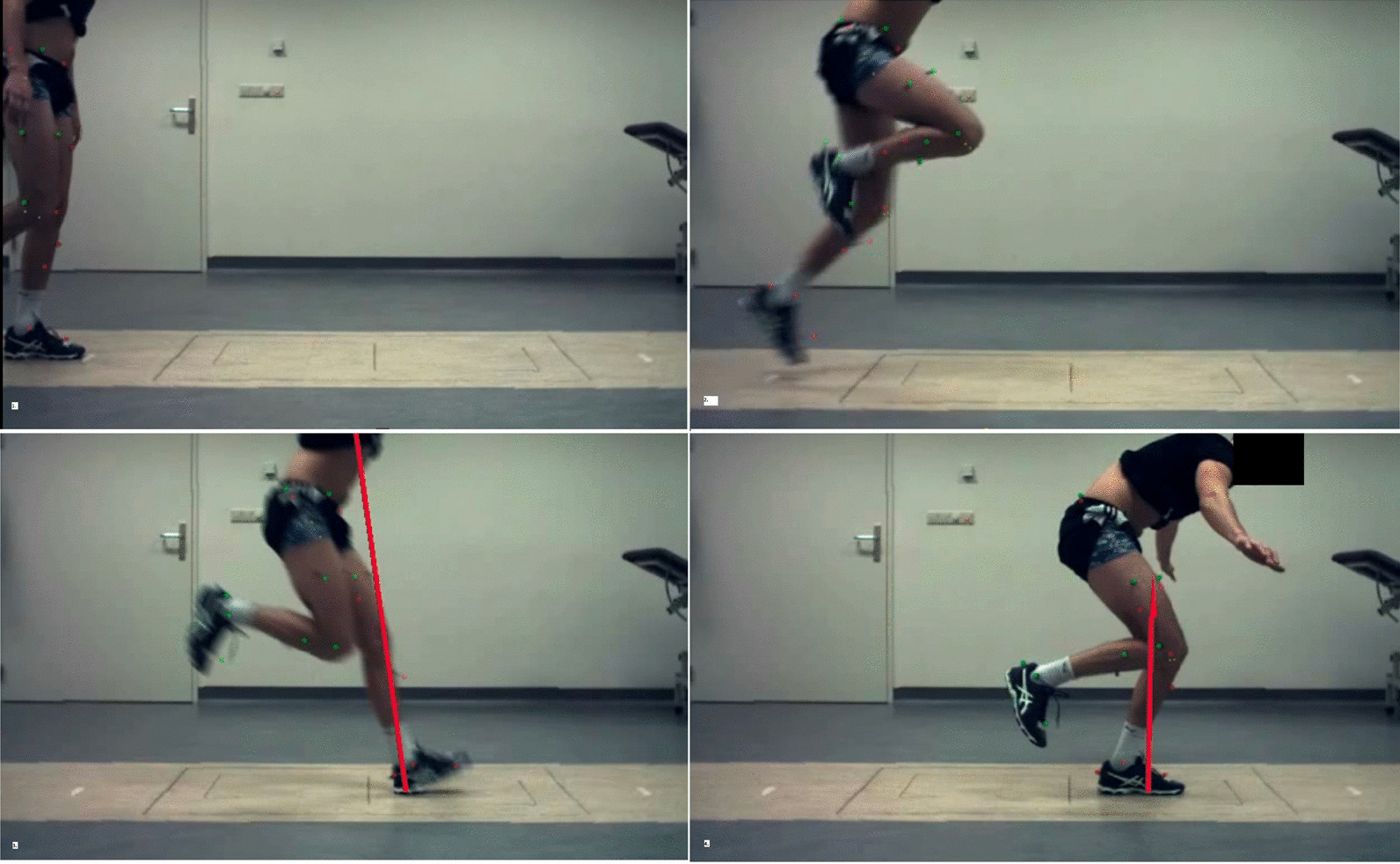
Example of a single-leg hop for distance

**Fig. 2:**
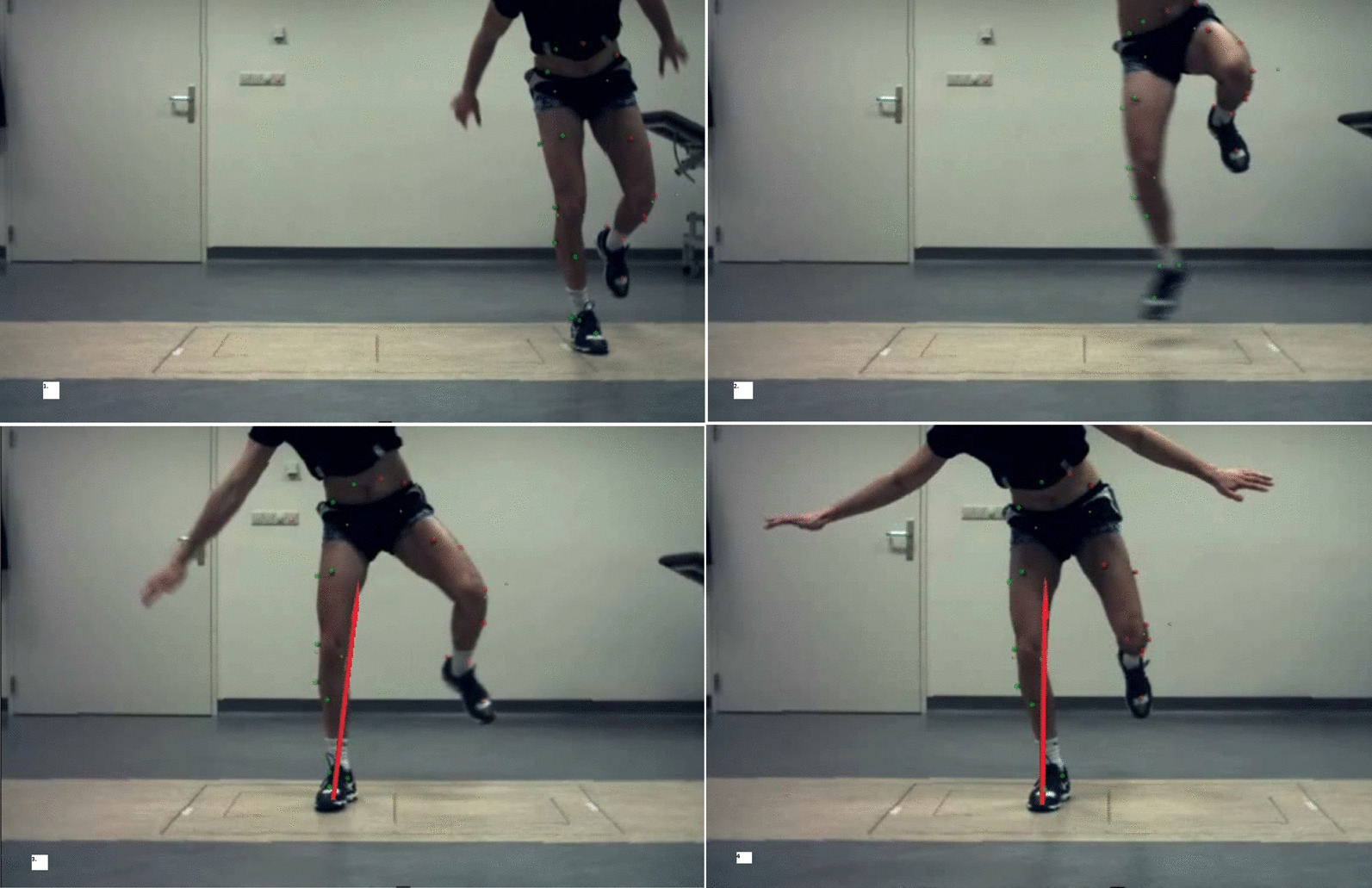
Example of a side jump

All subjects performed three tasks: (1) level walking at a self-selected pace; (2) a single-leg hop for distance (SLHD, maximum forward jump, jumping and landing on the same leg) (see Fig. 1); and (3) side jump (SJ, maximum sideways jump, jumping from and landing on the same leg) (see Fig. 2).

All jump trials were performed with hands in free motion and with sports shoes on. To familiarize subjects with the procedure and to make sure the entire foot landed on the force plate, subjects were asked to perform a dry run of the SLHD consisting of three practice trials. The median of the three practice hops was used to determine the starting distance from the force plates. For the side jump, leg length (greater trochanter tip to lateral malleolus tip) was used to determine the starting distance. Three approved trials per task were recorded for each knee to minimize the chances of data loss. Trials were approved when tasks were performed correctly (i.e. stable landing for at least 3 s), the entire foot landed on the force plate, and all markers were left in place. Approximately 13 months after the first trial—12 months after ACLR—the testing procedure was repeated.

### Data processing

The positions of the markers provided data to determine pelvic, femoral, tibial and foot segments. Using VICON Nexus software v2.8 and an additional custom MATLAB v9.7 script (The MathWorks Inc., Natick, MA, USA), three-dimensional angular displacements and translations in the knee joint were calculated. Data processing and analysis started at initial contact and continued for 200 ms. Initial contact was defined as the moment at which the vertical ground-reaction force (GRF) was > 5% of the body weight. All data were smoothed using the cross-validated quintic spline. Raw 3D marker position data were filtered using a low-pass frequency convolution filter of 10 Hz with zero lag. A maximum gap (temporary absence of marker identification) of ten frames was accepted to fill in using the software. If a trial contained gaps exceeding 2.5 ms smoothing of the data could not be performed and was therefore rejected. If at least two successful trials were available for a kinematic variable, the variable was included in the analysis. Kinematic variables quantified and included were: maximum knee flexion, maximum knee extension, maximum knee valgus, maximum knee varus, ATT, rTR and knee flexion moment. Knee flexion moment was calculated from the GRF vector and its lever arm to the centre of the knee of the stance leg. To quantify ATT and knee angles, two coordinate systems were reconstructed in the tested knee using the customized MATLAB script based on the method of Boeth et al. [24]. One system was reconstructed in the femoral segment (parent system) and one in the tibial segment (child system). The motion of each coordinate system was consistent with the movement of the respective segment. ATT was quantified in millimetres using the relative movement of the centre of rotation of the tibial coordinate system relative to the centre of rotation of the femoral coordinate system in the local tibial coordinate system. Tibial rotation was quantified by the angle between the two axes of rotation, as described by Keizer and Otten [23]. Flexion/extension, varus/valgus angles were obtained using scalar products as in the equations explained by Robertson et al. [25].

### Statistical analysis

Statistical analysis was performed using SSPS (v23; IBM Corp, Armonk, NY, USA). Since we had a small sample size, determining the distribution of the rTR was important for choosing appropriate statistical tests. A Shapiro–Wilk test was performed and did not show evidence of non-normality. Based on this outcome, and after visual examination of the QQ plot, we decided to use parametric tests. Means were calculated for each subject over the trials to obtain one value for each kinematic parameter per task. If at least two successful trials were available for a kinematic variable, the variable was included in analysis. To compare means of a kinematic variable a paired t-test was used with a significance level of p < 0.05. Three comparisons were made regarding the means of all kinematic data:

- Comparison of the pre-operative ACL-deficient knee vs. the post-operative ACL reconstructed knee (different time, same knee)
- Comparison of the pre-operative ACL-deficient knee vs. the pre-operative contralateral ACL-intact knee (same time, different knee)
- Comparison of the post-operative ACL-reconstructed knee vs. the post-operative contralateral ACL-intact knee (same time, different knee)

## Results

A total of 394 subjects with ACL injury were screened for participation in the study. Fifty-seven subjects met the inclusion and exclusion criteria and were asked to participate in the study. Ten subjects provided informed consent and were included in the study. All subjects underwent pre-rehabilitation as described before. Six males and four females remained and completed the primary testing procedures. At follow-up, one year after surgery, seven subjects participated (n = 7), as one subject had sustained a re-rupture (four months after reconstruction, due to a new trauma) and two subjects were lost to follow-up as they moved away from the Groningen region. The first measurements from the subjects lost to follow-up were included only in the pre-operative analyses comparing ACL deficient knees to the contra-lateral intact knees.

The patient who re-tore its ACL displayed less range of tibial rotation in both knees during level walking, compared to the group mean. During high-demand activities no major differences regarding rTR were found. The rTR for the subject with the re-tear of the ACL were as follows for the ACL deficient knee: level walking 6.9 (SD 1.1) degrees, SLHD 16.2 (SD 0.5) degrees and SJ 15.4 (SD 0.9) degrees. For the ACL intact knee the rTR was 10.6 (SD 0.2) degrees during level walking, 25.7 (SD 2.6) degrees during the SLHD and 22.8 (SD 3.2) degrees during the SJ.

Patient characteristics are presented in Table 1. No additional injuries to the menisci or cartilage were observed during surgery. No post-operative complications were reported. The mean distances for the SLHD were 105 cm (SD 33) for the ACL-deficient knees and 131 cm (SD 28) for the contralateral intact knees pre-operatively (significant difference, p = 0.01). One year after ACLR the SLHD was 115 cm (SD 50) for the ACL-reconstructed knees and 124 cm (SD 42) for the contralateral intact knees (non-significant difference, p = 0.11). A mean limb symmetry index for the SLHD test of 88% was achieved one year post-operatively. Four out of seven participating subjects had returned to sports activities 12 months post-operatively, three of them at their pre-injury level, based on participants reports.

**Table 1:** Patient characteristics (n = 10) and timeline

	Mean (SD)
Age	24 (4.4) years
Total body length	184 (10) cm
Total body weight	81.3 (8.9) kg
Body mass index	24.0 (2.1) kg/m^2^
Dominant leg injured	8 out of 10
Injury to first test interval	3.2 (1.2) months
Injury to surgery interval	4.6 (2.5) months
Surgery to second test interval (n = 7)	11.7 (1.9) months
First to second test interval (n = 7)	13 (1.1) months

### Kinematic outcome

During the first test 1080 values were acquired (ten subjects, two knees, six variables, three trials for walking, three trials for SLHD and three trials for side jump). A total of 50 values had to be discarded due to technical errors (4.6%, n = 10 in normal walking, n = 27 in SLHD, n = 13 in side jump) which were evenly distributed over the subjects. Seven participants performed the second test, leading to acquisition of 756 values, 30 of which had to be discarded due to technical errors (3.9% n = 18 in normal walking, n = 12 in SLHD, n = 0 in side jump). No variables had to be discarded due to missing data.

A significant difference between mean rTR in ACL-deficient knees compared to ACL-reconstructed knee was shown during the side jump. During all functional tests, a greater rTR was demonstrated after ACL reconstruction than shortly after ACL injury. This difference was only significant during the side jump (18.2 vs. 15.1, p = 0.04). The same trend was seen during level walking and the SLHD, but these differences in rTR were not significant. These results are displayed in Table 2; the values represent the data from the seven subjects who were available for both pre-operative and post-operative measurements. Before reconstruction, as shown in Table 3, rTR was smaller in ACL-deficient knees than in ACL-intact knees, although this difference was not significant.

**Table 2:** Mean range of tibial rotation for ACL-deficient and ACL-reconstructed knees (same knee, different timepoint) during level walking, SLHD and side jump

Range of tibial rotation (degrees (SD))			
	ACL-deficient	ACL-reconstructed	P-value†
Level walking	13.0 (2.2)	14.1 (3.9)	0.38
SLHD	16.3 (5.0)	17.4 (4.0)	0.39
Side Jump	15.1 (5.3)	18.2 (4.7)	0.04*

N = 7

SLHD = single-leg hop for distance, SD = standard deviation

^†^ Results of paired t-test comparing means of ACL-deficient and ACL-reconstructed knees

*indicates a significant result

**Table 3:** Mean range of tibial rotation for ACL-deficient and ACL-intact knees, both tested within three months after ACL injury, during level walking, SLHD and side jump

Range of tibial rotation (degrees (SD))			
	ACL-deficient	ACL-intact	P-value†
Level walking	13.7 (4.1)`	16.4 (5.6)	0.21
SLHD	16.9 (3.7)	19.4 (5.5)	0.21
Side Jump	16.6 (5.8)	20.7 (3.6)	0.08

N = 10

SLHD = single-leg hop for distance, SD = standard deviation

^†^Results of paired t-test comparing means of ACL-deficient and ACL-reconstructed knees

After reconstruction a significant difference in rTR between ACL-reconstructed and contralateral ACL-intact knees was found, as shown in Table 4: a significantly smaller rTR was observed in ACL-reconstructed knees compared to contralateral ACL-intact knees during all high-demand functional tests.

**Table 4:** Mean range of tibial rotation for ACL-reconstructed and ACL-intact knees, both tested one year after ACLR, during level walking, SLHD and side jump

Range of tibial rotation (degrees (SD))			
	ACL-reconstructed	ACL-intact	P-value†
Level walking	14.1 (3.9)	16.8 (4.6)	0.09
SLHD	17.4 (4.0)	22.8 (4.3)	0.01*
Side Jump	18.2 (4.7)	22.8 (5.6)	0.03*

N = 7

SLHD = single-leg hop for distance, SD = standard deviation

^†^Results of paired t-test comparing means of ACL-deficient and ACL-reconstructed knees

*indicates a significant result

**Fig. 3:**
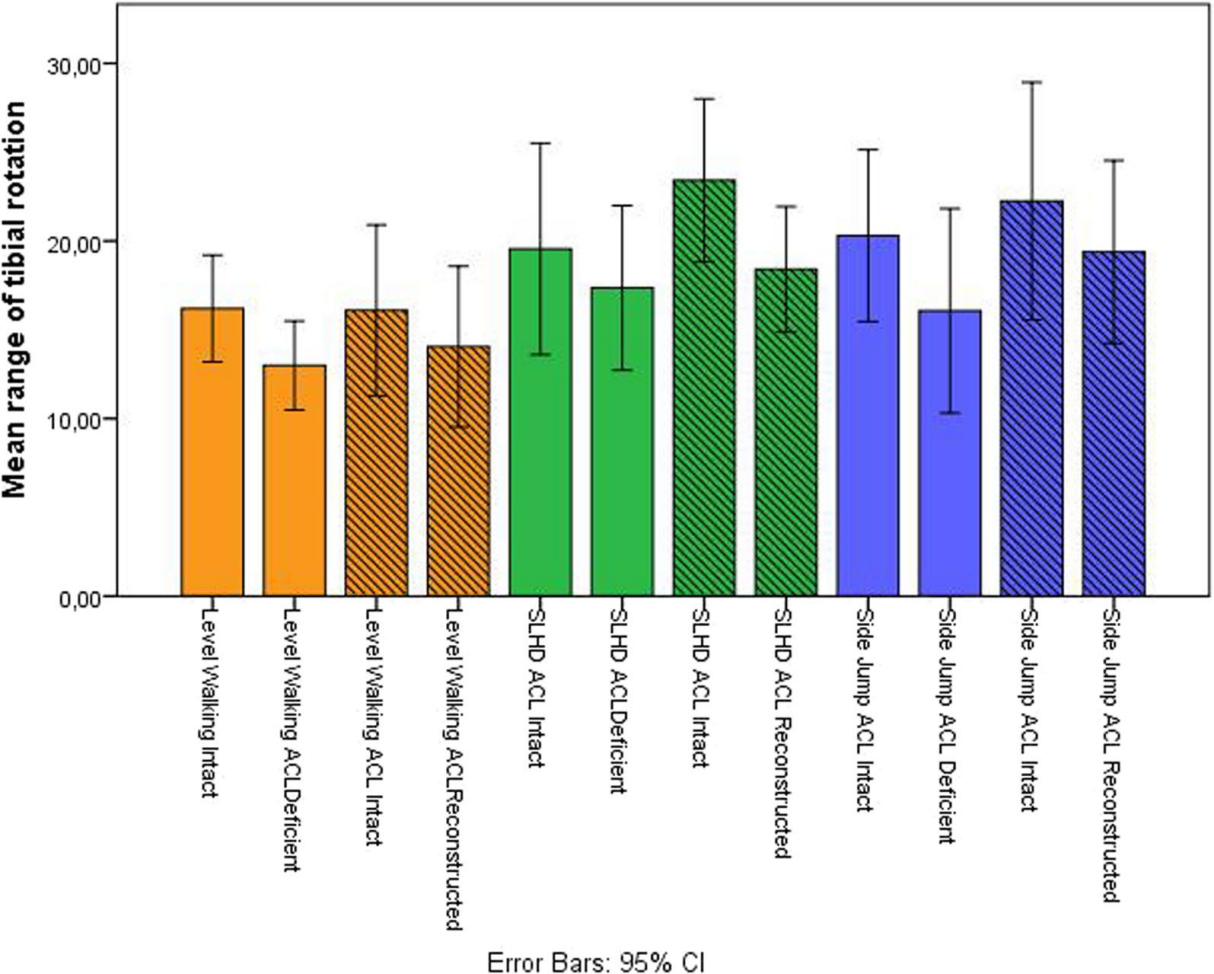
A bar chart illustrating mean rTR with a 95% confidence interval in ACL deficient knees, ACL intact knees both pre-and post-operative and ACL reconstructed knees during level walking, the SLHD and the side jump. Orange bars represent data obtained from level walking. Green bars represent data from a single leg hop for distance and blue bars represent data from a side jump. Bars with diagonal lines represent data from measurements one year after ACL reconstruction whereas bars without lines represent data from the pre-operative measurements, within 3 months after ACL injury

Figure 3 is a graphical representation of the results displaying mean rTR in ACL deficient, ACL intact and ACL reconstructed knees during the three different tasks.

The Additional file 1 shows an overview of the means of maximum knee flexion, maximum knee extension, maximum knee valgus, maximum knee varus, knee flexion moment and maximum ATT. No significant difference was seen in maximum knee flexion, maximum knee extension, maximum knee valgus, maximum knee varus or knee flexion moment during the SLHD and side jump between ACL-deficient and contralateral ACL-intact knees. During level walking ACL-deficient knees showed significantly less maximum knee extension than contralateral intact knees (5.5° vs. 3.5°, p = 0.02). This difference became apparent towards toe-off and not on initial contact.

ACL-reconstructed knees showed more maximum knee flexion (60.7° vs. 53.0°, p = 0.03) and less maximum knee extension (22.8° vs. 19.4°, p = 0.03) during the SLHD compared to the ACL-deficient knees. During level walking ACL-reconstructed knees showed less maximum knee flexion than contralateral ACL-intact knees (41.1° vs. 43.6°, p = 0.04).

During the SLHD the knee flexion moment was 5–6 times higher compared to level walking and 3 times higher compared to the side jump. There was no significant difference in the generated knee flexion moment between the injured and contralateral intact knees. See Additional file 1.

## Discussion

The main finding of our study was that, when measuring rTR in patients with a subacute ACL tear, a decrease in rTR compared to the contralateral knee was observed. Furthermore, one year after ACLR the rTR remained less than the contralateral knee. A combination of altered muscular contraction patterns and landing strategies may be responsible for these findings, rather than the result of the ACLR.

We observed a greater rTR during high-demand activities than during low-demand activities. During the hop tests the knees were exposed to a knee flexion moment six times higher than during level walking (Additional file 1). The hop tests have thus been a way of presenting a biomechanical challenge as well as a psychological one, in which fear of new injury may also have played an important role. Psychological factors like kinesiophobia, self-efficacy and fear of re-injury have been determined as important in ACL rehabilitation [26]. By asking subjects to perform a complex high-demand task, the effects of potentially deployed compensatory mechanisms become measurable. Hypothetically, a compensatory mechanism including altered muscular contraction may explain our findings, both before and after surgery. The exact mechanism of compensation cannot be determined based on our results, but as increased hamstring muscle activity can reduce anterior tibial translation [27], and increased activity of the m. biceps femoris in collaboration with the iliotibial band can be responsible for counteracting the rotational forces, we hypothesise that even shortly after the injury a neuromuscular adaptation in patients with ACL-deficient knees may occur. Neuromuscalar control is the result of a complex integration of vestibular, somatosensory and visual stimuli and is affected by situational awareness, arousal and attention [28]. Muscular contraction is continuously fine-tuned on the anticipated demands of the knee to preserve joint equilibrium and stability. After ACL injury, it is suggested that the central nervous system relies more on visual feedback and spatial awareness, as the biomechanical feedback is disturbed [28]. Accordingly, previous studies showed that muscle activation patterns of patients with an ACL-injured knee and after an ACLR are modified compared to healthy knees [27, 29–31]. This ‘increased stiffening’ strategy as compensation for perceived instability has been proposed before; by altering jumping technique (less high and less far), and landing technique (less knee flexion), more stiffness is introduced in the knee joint [29]. Altered landing techniques were also demonstrated by Keizer et al. in healthy subjects with intact ACLs but with higher knee laxity [32]. In our study we also observed less maximum knee flexion in ACL-deficient knees compared to ACL-reconstructed knees, but there were no or only very small differences between the affected and the contralateral ACL-intact knees in terms of maximum knee flexion. When muscular compensation and, through this, altered landing kinematics indeed are a valid explanation for our observations, this mechanism would prevent symptomatic knee laxity in chronic ACL deficiency too. Yet, in the acute phase, shortly after a traumatic event, fear of re-injury may contribute to increased stiffening as well [33], and as the fear diminishes over time this can cause the knee laxity to become clinically apparent. We therefore hypothesise that a combination of an altered landing strategy, altered muscular contraction patterns and fear of re-injury can lead to a smaller rTR in ACL-affected knees.

Our results differ from other study results regarding rTR in ACL deficiency. Cadaveric studies and studies in passive situations have shown that rupture of the ACL allows more, passive, rotation of the tibia [34]. An increased rTR in ACL deficiency compared to healthy knees has also been shown during functional yet low to moderate demand tasks [11, 12, 14–16]. Results from these studies are shown in Table 5. As seen in Table 5, we measured a smaller rTR after ACLR compared to the contralateral intact knee, as did other authors.

**Table 5:** Overview of reported values for range of tibial rotation using motion capture systems

Author	Task performed	ACL status	Range of tibial rotation
Zee (current study)	SLHD	Intact	19.4°
		Deficient	16.9°
		SB reconstruction	18.4°
Cheng [16]	Jump off platform, pivot 90°	Intact	6.7°
		Deficient	13.5°
		SB reconstruction	7.8°
		DB reconstruction	7.5°
Lam [8]	Jump off platform, pivot 90°	Deficient	12.6°
		SB reconstruction	8.9°
Misonoo [11]	Jump off platform, pivot 45°	Intact	20.8°
		SB reconstruction	21.4°
		DB reconstruction	22.0°
Ristanis [12]	Step off stairs, pivot 90°	Intact	19.0°
		SB reconstruction	18.6°
Tsahouras [14]	Standing, pivoting 60°	Intact	13.9°
		Deficient	15.1°
		SB reconstruction	13.4°
		DB reconstruction	13.4°
Tsahouras [15]	Step off stairs, pivot 60°	Intact	14.2°
		Deficient	15.3°
		SB reconstruction	12.7°
		DB reconstruction	13.9°

ACL = anterior cruciate ligament, SLHD = single-leg hop for distance, SB = single bundle, DB = double bundle

Two key features of our study are distinctly different from previous research, which could explain the differences found in the ACL-deficient knees: we performed our tests within three months after injury and used high-demand tasks. Firstly, time since injury is an important aspect when measuring rTR in ACL-deficient knees, as it seems that in the acute phase subjects are able to limit rTR. Testing more than one year after the injury, both Cheng and Tsarouhas found a greater rTR in ACL-deficient knees compared to contralateral intact knees [14–16]. Miyaji et al., on the other hand, studied ACL-deficient subjects with a median time since injury of 10 weeks (range 3.3–450 weeks, mean 47 weeks) and observed a smaller rTR in the ACL-deficient knees compared to uninjured contralateral knees during a wide based squat [35]. This is in accordance with our findings. These findings emphasise the influence of *time since injury* on knee kinematics after ACL injury. In the acute setting, subjects exhibit different jumping strategies during activities (protective secondary to recent trauma) than weeks later. Weeks later, the secondary stabilizers of the knee may have stretched due to the altered mechanical load in the absence of the ACL. This may lead to an alteration of kinematics of the knee with the passage of time.

Our study provides additional information for the debate on rTR due to a new measurement moment, namely in the acute phase after an ACL rupture. This also puts the post-operative measurements in a different light. Ristanis and Tsarouhas demonstrated that, after ACLR, rTR is smaller compared to contralateral-intact knees [12, 14, 15]. This has been attributed to overconstrainment of the graft [11]. It is questionable whether the reduced rTR post-operatively can be attributed to overtightening of the graft, as a smaller rTR was also found in ACL-deficient knees before ACLR (and even smaller compared to post-ACLR). Again, perhaps altered landing strategy, altered muscular contraction patterns and fear of re-injury should be taken into account more. Also, it has been shown in dogs that intact sensory nerves around the knee, probably by influencing protective muscular reflexes, play an important role in preventing the acutely unstable knee from rapid breakdown [36]. Our study may indicate that these strategies have already started at the initial evaluation within 3 months after injury and are indelible by one year after reconstruction.

Secondly, our study differs from previous research in terms of the used functional tasks: our subjects performed both low and high-demand functional tasks as opposed to previously reported low-to-moderate-demand functional tasks. Our results of rTR during level walking (low demanding) are comparable to earlier reports, both pre- and post-operatively [37]. The rTR has not been previously measured using a motion capture system while the subjects were performing a SLHD or a side-jump. A hop test is a complex, high-demand task in which a lot of force is generated in the knee, and can also induce fear of injury. As seen in the Additional file 1 over 5–6 times more knee flexion moment is applied to the knee during the SLHD compared to level walking. This is therefore likely the best functional test to mimic sports activities, but in a safe clinical setting. We recommend using hop tests when measuring rTR in the context of ACL injury or after ACLR. Plus, the uniform use of hop tests ensures that studies can be compared.

In our study a return to sports rate of four out of seven (57%) was achieved 12 months after ACL reconstruction which is representative for the recreational athlete according to the literature [38]. This emphasizes the lengthy recovery after ACLR. Return to sports within 12 months after ACLR may not be a realistic goal in all patients undergoing ACLR and pre-operative counselling should take this into account. Rehabilitation programmes that include perturbation training, agility training, vision training and sport specific skill training are essential after ACL injury and reconstruction [28]. The neuromuscular system adapts to unaccustomed loads, also known as overload [39]. Therefore for optimization of the neuromuscalar system, changes in volume and intensity of training is needed, as without this, there is no need for the neuromuscular system to improve [39]. A periodized rehabilitation program aims to optimize the principle of overload. Rehabilitation planned according to the periodization concepts could allow better integration of the needs of the patients to return to sport [39]. When paying special attention to postural control and proprioceptive function of the knee during rehabilitation, significant smaller knee abduction moments were observed compared to traditional rehabilitation programmes, indicating better knee stability [40].

### Study strengths and limitations

A strength of our study is the fact that we measured rTR in contrast to absolute values of rotation. Other papers focusing on absolute values of tibial rotation showed that ACL-deficient subjects tend towards a more externally rotated tibia [41]. It is difficult to repeat the measurements with this method: subsequent measurements with marker placement in a slightly different position with respect to bony landmarks will lead to major differences [42], hence in a longitudinal study design the use of absolute values of rotation is not preferred. A relative outcome such as range of rotation is more reliable and allows for repeatable measurements over time.

In our study we used the contralateral intact knee as a comparison. There is sparse literature available that shows that the contralateral intact knee also shows an altered movement pattern after an ACL injury. This has been particularly demonstrated in the post-operative phase during hop testing [43]. Whether this occurs immediately after the injury is unclear. It also has been shown that abnormal geometrical characteristics in the knee, that may be present bilaterally, pose a risk factor for ACL injury [44]. Whether and how this affects the kinematics of the knee is unclear. We can compare our results to available literature regarding healthy knees. Liu et al. studied knee kinematics during walking and running in healthy subjects [45]. Although the study of Liu et al. use a different method to measure range of tibial rotation it can serve as a basis to compare our results to. Liu showed a rTR of 14.0 ± 4 degrees during walking at 3 km/h and 15.5 ± 4.1 degrees during walking at 5 km/h. These results seem comparable to our results during level walking, although we have not recorded the walking pace of our subjects. Also, leg dominance may be a potential confounder. In our population 8 out of 10 ACL injured knees were dominant legs. Whether and how this influenced our results is unclear.

Small sample size is an issue that has to be taken into account when evaluating our results. The narrow inclusion and exclusion criteria are mainly responsible for the small sample size. Subjects with concomitant injury were excluded as injury to the menisci and anterolateral structures of the knee are known to influence degree of tibial rotation [21]. This narrows the number of eligible subjects.

As some subjects with a recent ACL injury may have been reluctant to participate in the study after being informed on the hop test, a certain amount of selection bias may be present. Although the inclusion criteria were strictly based on the dutch guideline for ACL injury, the motivation for definite participation could have been subject to individual variables like available time or fear for re-injury.Subjects with a greater feeling of giving way may not have participated.

Despite these limitations, in these patients we have objectively measured that rTR in the ACL-deficient knee is not greater than in the contralateral ACL-intact knee shortly after ACL injury. Further research is needed to elucidate why rTR is not higher or even lower in acute ACL injury. Up to now we have found no evidence to suggest that persistent increased rotational laxity hampers return to play after ACLR. Special attention to neuromuscular control, subjective knee function and psychological factors may help us better understand which factors play an important role in whether objective knee instability occurs, which ultimately may hamper return to sports rates. In this light, testing subjects in circumstances that replicate sport activities, i.e. using hoptests, is crucial.

## Conclusion

No increase in range of tibial rotation is shown in subacute ACL-injured knees compared to contralateralal intact knees during high-demand tasks. One year after ACL reconstruction, a smaller range of tibial rotation is observed compared to ACL-intact knees. Further research into altered motor control strategies and psychological factors like fear of re-injury could elucidate this unexpected phenomenon. We propose the use of hop tests as high-demand, complex tasks when evaluating range of tibial rotation both before and after ACL reconstruction.

## Supplementary Information

**Additional file 1**.** Appendix A**. Kinematic results.

## Data Availability

The datasets used and/or analysed during the current study are available from the corresponding author on reasonable request.

## Abbreviations

ACL: Anterior cruciate ligament
ACLR: Anterior cruciate ligament reconstruction
ATT: Anterior tibial translation
GRF: Ground reaction force
Hz: Herz
Nm: Newton-metre
MRI: Magnetic resonance imaging
ms: Milliseconds
rTR: Range of tibial rotation
SD: Standard deviation
SJ: Side Jump
SLHD: Single Leg Hop for Distance
UMCG: University Medical Center Groningen

## References

[1] Ardern CL, Taylor NF, Feller JA (2014). Fifty-five per cent return to competitive sport following anterior cruciate ligament reconstruction surgery: an updated systematic review and meta-analysis including aspects of physical functioning and contextual factors. Br J Sports Med.

[2] Fu FH, Bennett CH, Ma CB (2000). Current trends in anterior cruciate ligament reconstruction. Part II. Operative procedures and clinical correlations. Am J Sports Med.

[3] Chouliaras V, Ristanis S, Moraiti C (2007). Effectiveness of reconstruction of the anterior cruciate ligament with quadrupled hamstrings and bone-patellar tendon-bone autografts: an in vivo study comparing tibial internal-external rotation. Am J Sports Med.

[4] Christino MA, Vopat BG, Waryasz GR (2014). Adolescent differences in knee stability following computer-assisted anterior cruciate ligament reconstruction. Orthop Rev (Pavia).

[5] Christino MA, Vopat BG, Mayer A (2015). Stability outcomes following computer-assisted ACL reconstruction. Minim Invasive Surg.

[6] Debieux P, Carneiro M, de Queiroz AAB (2012). Sagittal and rotational knee stability following single- and double-bundle reconstruction of the anterior cruciate ligament: a randomized clinical trial. Eur Orthop Traumatol.

[7] King E, Richter C, Franklyn-Miller A (2018). Biomechanical but not timed performance asymmetries persist between limbs 9months after ACL reconstruction during planned and unplanned change of direction. J Biomech.

[8] Lam MH, Fong DT, Yung PS (2011). Knee rotational stability during pivoting movement is restored after anatomic double-bundle anterior cruciate ligament reconstruction. Am J Sports Med.

[9] Lee S, Kim H, Jang J (2012). Comparison of anterior and rotatory laxity using navigation between single- and double-bundle ACL reconstruction: prospective randomized trial. Knee Surg Sports Traumatol Arthrosc.

[10] Matic A, Petrovic Savic S, Ristic B (2016). Infrared assessment of knee instability in ACL deficient patients. Int Orthop.

[11] Misonoo G, Kanamori A, Ida H (2012). Evaluation of tibial rotational stability of single-bundle vs. anatomical double-bundle anterior cruciate ligament reconstruction during a high-demand activity - a quasi-randomized trial. Knee.

[12] Ristanis S, Giakas G, Papageorgiou CD (2003). The effects of anterior cruciate ligament reconstruction on tibial rotation during pivoting after descending stairs. Knee Surg Sports Traumatol Arthrosc.

[13] Takeda K, Hasegawa T, Kiriyama Y (2014). Kinematic motion of the anterior cruciate ligament deficient knee during functionally high and low demanding tasks. J Biomech.

[14] Tsarouhas A, Iosifidis M, Kotzamitelos D (2010). Three-dimensional kinematic and kinetic analysis of knee rotational stability after single- and double-bundle anterior cruciate ligament reconstruction. Arthroscopy.

[15] Tsarouhas A, Iosifidis M, Spyropoulos G (2011). Tibial rotation under combined in vivo loading after single- and double-bundle anterior cruciate ligament reconstruction. Arthroscopy.

[16] Cheng K, Chen W, Lee H (2012). Dynamic functional performance and kinematic analysis of the rotational patterns of single- versus double-bundle anterior cruciate ligament reconstruction. Formos J Musculoskelet Disord.

[17] Rivera-Brown AM, Frontera WR, Fontanez R (2022). Evidence for isokinetic and functional testing in return to sport decisions following ACL surgery. PM R.

[18] Hildebrandt C, Muller L, Zisch B (2015). Functional assessments for decision-making regarding return to sports following ACL reconstruction. Part I: development of a new test battery. Knee Surg Sports Traumatol Arthrosc.

[19] Kaplan Y, Witvrouw E (2019). When is it safe to return to sport after ACL reconstruction?. Rev Criteria Sports Health.

[20] Neeter C, Gustavsson A, Thomee P (2006). Development of a strength test battery for evaluating leg muscle power after anterior cruciate ligament injury and reconstruction. Knee Surg Sports Traumatol Arthrosc.

[21] Kittl C, El-Daou H, Athwal KK (2016). The role of the anterolateral structures and the ACL in controlling laxity of the intact and ACL-deficient knee: response. Am J Sports Med.

[22] Davis RB, Ounpuu S, Tyburski D (1991). A gait analysis data collection and reduction technique. Hum Mov Sci.

[23] Keizer MNJ, Otten E (2020). Technical note: sensitivity analysis of the SCoRE and SARA methods for determining rotational axes during tibiofemoral movements using optical motion capture. J Exp Orthop.

[24] Boeth H, Duda GN, Heller MO (2013). Anterior cruciate ligament-deficient patients with passive knee joint laxity have a decreased range of anterior-posterior motion during active movements. Am J Sports Med.

[25] Robertson D, Caldwell G, Hamill J (2013). Research Methods in Biomechanics.

[26] Ardern CL, Taylor NF, Feller JA (2013). Psychological responses matter in returning to preinjury level of sport after anterior cruciate ligament reconstruction surgery. Am J Sports Med.

[27] Shelburne KB, Torry MR, Pandy MG (2005). Effect of muscle compensation on knee instability during ACL-deficient gait. Med Sci Sports Exerc.

[28] Kakavas G, Malliaropoulos N, Pruna R (2020). Neuroplasticity and anterior cruciate ligament injury. Indian J Orthop.

[29] Hurd WJ, Snyder-Mackler L (2007). Knee instability after acute ACL rupture affects movement patterns during the mid-stance phase of gait. J Orthop Res.

[30] Klyne DM, Keays SL, Bullock-Saxton JE (2012). The effect of anterior cruciate ligament rupture on the timing and amplitude of gastrocnemius muscle activation: a study of alterations in EMG measures and their relationship to knee joint stability. J Electromyogr Kinesiol.

[31] Barcellona MG, Morrissey MC, Milligan P (2014). The effect of thigh muscle activity on anterior knee laxity in the uninjured and anterior cruciate ligament-injured knee. Knee Surg Sports Traumatol Arthrosc.

[32] Keizer MNJ, Hijmans JM, Gokeler A (2020). Healthy subjects with lax knees use less knee flexion rather than muscle control to limit anterior tibia translation during landing. J Exp Orthop.

[33] Trigsted SM, Cook DB, Pickett KA (2018). Greater fear of reinjury is related to stiffened jump-landing biomechanics and muscle activation in women after ACL reconstruction. Knee Surg Sports Traumatol Arthrosc.

[34] Zee MJM, Robben BJ, Zuurmond RG (2020). Effect of ACL reconstruction on range of tibial rotation: a systematic review of current literature and a recommendation for a standard measuring protocol. Orthop J Sports Med.

[35] Miyaji T, Gamada K, Kidera K (2012). In vivo kinematics of the anterior cruciate ligament deficient knee during wide-based squat using a 2D/3D registration technique. J Sports Sci Med.

[36] O'Connor BL, Visco DM, Brandt KD (1992). Neurogenic acceleration of osteoarthrosis. The effects of previous neurectomy of the articular nerves on the development of osteoarthrosis after transection of the anterior cruciate ligament in dogs. J Bone Joint Surg Am.

[37] Asaeda M, Deie M, Fujita N (2017). Gender differences in the restoration of knee joint biomechanics during gait after anterior cruciate ligament reconstruction. Knee.

[38] Ardern CL, Webster KE, Taylor NF (2011). Return to sport following anterior cruciate ligament reconstruction surgery: a systematic review and meta-analysis of the state of play. Br J Sports Med.

[39] Kakavas G, Malliaropoulos N, Bikos G (2021). Periodization in anterior cruciate ligament rehabilitation: a novel framework. Med Princ Pract.

[40] Papalia R, Franceschi F, Tecame A (2015). Anterior cruciate ligament reconstruction and return to sport activity: postural control as the key to success. Int Orthop.

[41] Zhang LQ, Shiavi RG, Limbird TJ (2003). Six degrees-of-freedom kinematics of ACL deficient knees during locomotion-compensatory mechanism. Gait Posture.

[42] McFadden C, Daniels K, Strike S (2020). The sensitivity of joint kinematics and kinetics to marker placement during a change of direction task. J Biomech.

[43] Hofbauer M, Thorhauer ED, Abebe E (2014). Altered tibiofemoral kinematics in the affected knee and compensatory changes in the contralateral knee after anterior cruciate ligament reconstruction. Am J Sports Med.

[44] Levins JG, Argentieri EC, Sturnick DR (2017). Geometric characteristics of the knee are associated with a noncontact ACL injury to the contralateral knee after unilateral ACL injury in young female athletes. Am J Sports Med.

[45] Liu R, Qian D, Chen Y (2021). Investigation of normal knees kinematics in walking and running at different speeds using a portable motion analysis system. Sports Biomech.

