# Supplementary material for: High-demand tasks show that ACL reconstruction is not the only factor in controlling range of tibial rotation: a preliminary investigation
Source: J Orthop Surg Res. 2023 Mar 13;18:194. doi: 10.1186/s13018-023-03639-2 (PMC10009984; doi:10.1186/s13018-023-03639-2)
Supplement: Supplementary file 1 — Additional file 1. Appendix A. Kinematic results. [file 13018_2023_3639_MOESM1_ESM.docx]

Appendix

P-value 3

P-value 2

P-value 1

| Kinematic/Kinetic variable | Pre-operative | |  | Post-operative | |  |  |
| --- | --- | --- | --- | --- | --- | --- | --- |
|  | ACL-intact | ACL-deficient | P-value 1 | ACL-intact | ACL-reconstructed | P-value 2 | P-value 3 |
| **Level Walking** | | | | |  |  |  |
| Max knee flexion, in degrees (SD) | 42.7 (7.2) | 41.4 (6.6) | 0.33 | **43.6 (4.5)** | **41.1 (4.9)** | 0.30 | **0.04*** |
| Max knee extension, in degrees (SD) | **3.5 (2.2)** | **5.5 (3.5)** | **0.02*** | 4.7 (2.3) | 6.0 (2.2) | 0.81 | 0.13 |
| Max knee valgus, in degrees (SD) | 4.4 (1.8) | 4.4 (1.7) | 1.00 | 5.3 (2.2) | 4.7 (1.9) | 0.92 | 0.46 |
| Max knee varus, in degrees (SD) | 1.1 (1.2) | 0,9 (0.5) | 0.75 | 0.8 (0.9) | 1.0 (0.8) | 0.47 | 0.48 |
| Knee flexion moment, in Nm (SD) | 0.8 (0.3) | 0.9 (0.3) | 0.09 | 0.9 (0.3) | 0.8 (0.2) | 0.49 | 0.49 |
| Max ATT, in mm (SD) | 6.6 (3.0) | 4.6 (4.8) | 0.06 | 6.2 (6.6) | 4.4 (6.6) | 0.25 | 0.25 |
| Rang of tibial rotation | 16.4 (5.6) | 13.7 (4.1) | 0.21 | 16.8 (4.6) | 14.1 (3.9) | 0.38 | 0.09 |
| **Single Leg Hop for Distance** | | | | |  |  |  |
| Max knee flexion, in degrees (SD) | 57.5 (9.5) | **53.0 (6.7)** | 0.13 | 61.4 (10.5) | **60.7 (10.4)** | **0.03*** | 0.75 |
| Max knee extension, in degrees (SD) | 19.4 (4.0) | **19.4 (2.7)** | 0.98 | 20.4 (6.3) | **22.8 (3.5)** | **0.03*** | 0.30 |
| Max knee valgus, in degrees (SD) | 9.1 (4.5) | 6.3 (3.8) | 0.10 | 9.9 (4.9) | 7.2 (1.8) | 0.27 | 0.15 |
| Max knee varus, in degrees (SD) | -0.3 (0.9) | 1.5 (3.8) | 0.16 | 0.3 (2.4) | 1.1 (1.8) | 0.60 | 0.50 |
| Knee flexion moment, in Nm (SD) | 5.2 (0.8) | 6.2 (1.4) | .14 | 5.9 (1.9) | 5.2 (1.0) | 0.50 | 0.50 |
| Max ATT, in mm (SD) | 13.4 (7.2) | 10.1 (5.4) | 0.89 | 12.7 (3.4) | 12.2 (8.5) | 0.82 | 0.82 |
| Range of tibial rotation | 19.4 (5.5) | 16.9 (3.7) | 0.21 | **22.8 (4.3)** | **17.4 (4.0)** | 0.39 | **0.01*** |
| **Side Jump** | | | | |  |  |  |
| Max knee flexion, in degrees (SD) | 49.1 (16.4) | 51.8 (7.2) | 0.54 | 56.7 (7.4) | 56.1(9.8) | 0.15 | 0.77 |
| Max knee extension, in degrees (SD) | 25.2 (5.8) | 29.6 (3.7) | 0.06 | 28.3 (7.3) | 29.7 (7.8) | 0.74 | 0.44 |
| Max knee valgus, in degrees (SD) | 10.3 (5.2) | 7.9 (4.9) | 0.17 | 11.3 (6.4) | 8.6 (3.0) | 0.11 | 0.19 |
| Max knee varus, in degrees (SD) | -2.0 (3.4) | 0.0 (3.7) | 0.10 | -2.5 (4.0) | -0.3 (2.1) | 0.43 | 0.10 |
| Knee flexion moment, in Nm (SD) | 1.9 (0.6) | 2.4 (0.9) | .27 | 2.2 (0.7) | 1.7 (0.5) | 0.14 | 0.14 |
| Max ATT, in mm (SD) | 7.7 (5.8) | 6.7 (5.5) | 0.37 | 7.6 (5.5) | 8.9 (7.6) | 0.51 | 0.51 |
| Range of tibial rotation | 20.7 (3.6) | **16.6 (5.8)** | 0.08 | **22.8 (5.3)** | **18.2 (4.7)** | **0.04*** | **0.03*** |
| P-value 1 = results of paired t-test comparing means of ACL-deficient and contralateral ACL-intact knees  P-value 2 = results of paired t-test comparing means of ACL-deficient and ACL-reconstructed knees  P-value 3= results of paired t-test comparing means of ACL-reconstructed and contralateral ACL-intact knee  ATT = Anterior Tibial Translation Nm = newton-metre, SD = standard deviation | | | | | | | |
